# The Application of iPSCs in Tumour Immunotherapy

**DOI:** 10.1017/erm.2025.10006

**Published:** 2025-08-11

**Authors:** Peinan Chen, Jian Gao, Jianing Feng, Hongfei Tao, Yongkui Yu, Yijing Li, Jinlong Liu, Shuangshuang Lu, Wei Wang

**Affiliations:** 1 Department of Thoracic surgery, The Affiliated Cancer Hospital of Zhengzhou University & Henan Cancer Hospital, Zhengzhou, China; 2 National Center for International Research in Cell and Gene Therapy, Sino-British Research Centre for Molecular Oncology, School of Basic Medical Sciences, Tianjian Advanced Biomedical Laboratory, Academy of Medical Sciences, Zhengzhou University, Zhengzhou, China

**Keywords:** induced pluripotent stem cell, iPSC-derived immune cells, iPSC-derived tumour vaccine, opportunities and challenges, tumour immunotherapy

## Abstract

**Background:**

Tumour immunotherapy holds great promise as a treatment for cancer, which ranks as the second highest cause of mortality worldwide. This therapeutic approach can be broadly categorized into two main types: active immunotherapy and passive or adoptive immunotherapy. Active immunotherapy, such as cancer vaccines, stimulates the patients’ immune system to target tumour cells. On the other hand, adoptive immunotherapy involves supplying in vitro activated immune cells, such as T cells, natural killer cells and macrophages, to the patient to combat the tumour. Induced pluripotent stem cells are extensively utilized in both active and adoptive tumour immunotherapy due to their pluripotency and ease of gene editing. They can be differentiated into various types of immune cells for direct cancer treatment and can also function as tumour vaccines to elicit an immune response against the tumour. Importantly, iPSCs can be leveraged to develop off-the-shelf allogenic immunotherapy products.

**Conclusion:**

This article provides a comprehensive review of the application of iPSCs in tumor immunotherapy, along with a discussion of the opportunities and challenges in this evolving field.

## Introduction

In 2006, Yamanaka’s team from Kyoto University made a significant breakthrough by demonstrating the induction of pluripotent cells from mouse fibroblasts through the retroviral introduction of four transcription factors – Oct3/4, Sox2, c-Myc and Klf4 – collectively known as Yamanaka factors (Ref. 1). These reprogrammed cells, termed induced pluripotent stem cells (iPSCs), exhibit many similar characteristics to embryonic stem cells (ESCs) but without the ethical controversies associated with the latter. Furthermore, iPSCs offer two additional key advantages for their practical application: high pluripotency and facile gene-editing capabilities. Their high pluripotency enables their differentiation into various target cell types, while their amenability to gene editing makes them valuable for disease modelling and investigating the function of mutant genes. As a result, iPSCs hold great promise for diverse applications in regenerative medicine, including tissue and cell replacement, disease modelling, understanding of pathogenesis, development and selection of therapeutic drugs and even organ synthesis (Ref. 2). This breakthrough in iPSC technology has ushered in a new era of stem cell therapy, culminating in the awarding of the 2012 Nobel Prize in Physiology or Medicine to Shinya Yamanaka and John Gurdon for their pioneering discovery that mature cells can be reprogrammed to become pluripotent (Ref. 3).

**Figure 1.**
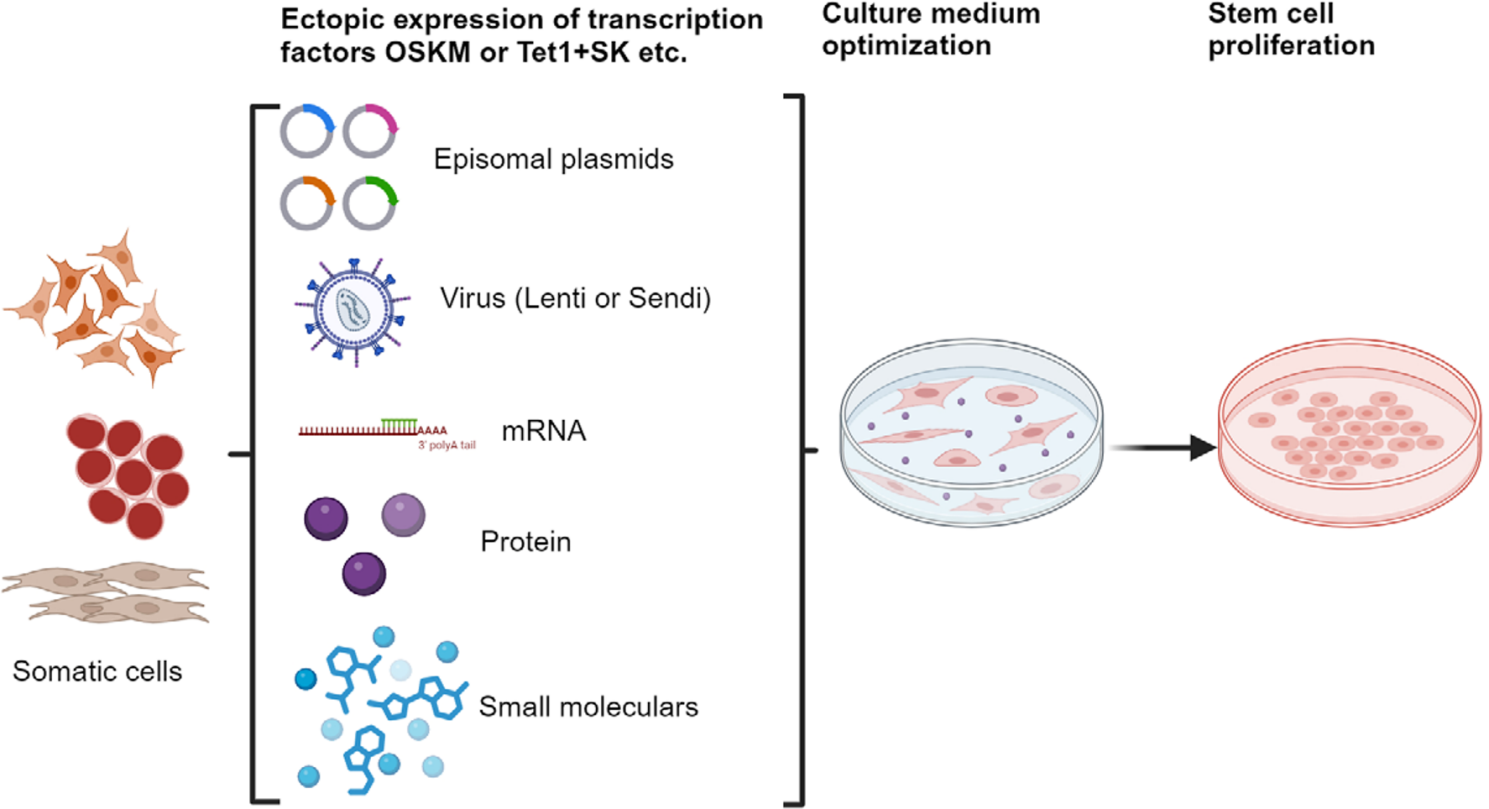
Schematic presentation of the methods and process for iPSCs production.

The development of iPSCs has sparked significant interest due to their potential applications. To enhance the safety and efficacy of this technology, extensive efforts have been directed towards replacing the oncogenes c-Myc and Klf4 with non-viral small molecules or proteins, thereby avoiding the introduction of exogenous genes (Ref. 4). As a result, a variety of commercially available culture media have been established to facilitate the generation of iPSCs from diverse somatic cell types, as illustrated in Figure 1, paving the way for clinical applications.

Tumours represent the second leading cause of death globally (Ref. 5). The conventional approaches of surgery, chemotherapy and radiotherapy have long been the mainstays of cancer treatment (Ref. 6), yet their efficacy in advanced or recurrent tumours remains limited. Both basic and clinical research have underscored the pivotal role of the tumour microenvironment, comprising cancerous, stromal and immune cells, in driving cancer progression. Recent studies have demonstrated the potential of immunotherapies in targeting the tumour microenvironment and enhancing the clinical management of oral cancer (Ref. 7). Immunotherapy has emerged as a promising avenue for the treatment of advanced or recurrent tumours, aiming to harness the host immune system to confer passive or active immunity against malignant tumours. Passive immunity involves the *ex vivo* activation and transfer of immune cells to patients, exemplified by chimeric antigen receptor (CAR)-T therapy, while active immunity entails *in vivo* activation of patients’ own immune cells through exposure to a foreign antigen, as seen in cancer vaccines.

**Figure 2.**
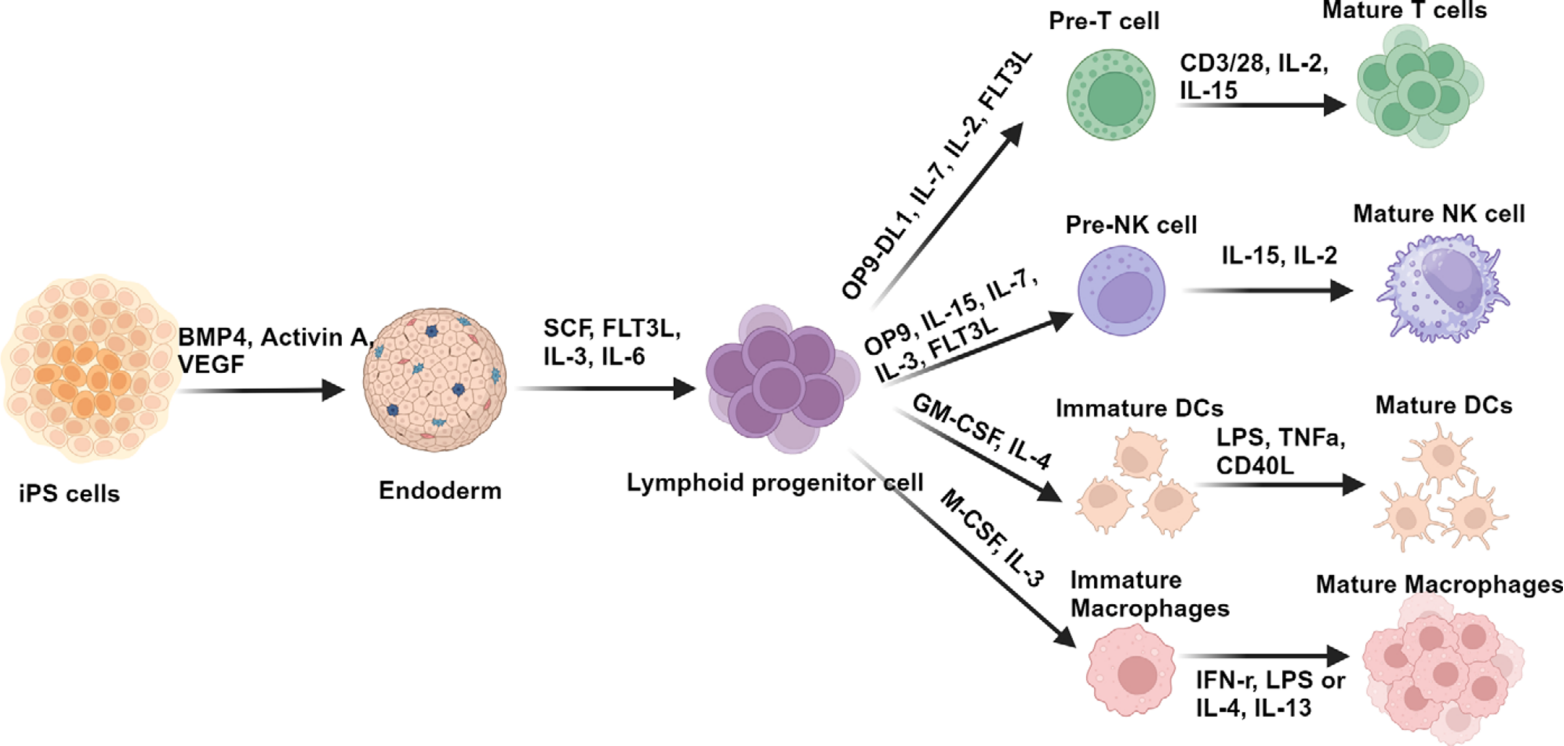
Schematic presentation of iPSCs differentiation into immune cells.

The use of iPSCs in tumour immunotherapy is a promising area of research to conquer the challenges associated with obtaining sufficient numbers and the high cost of autologous immune cells. iPSCs possess pluripotency, allowing them to differentiate into various immune cells, including T cells, natural killer (NK) cells, macrophages and dendritic cells (DCs), as illustrated in Figure 2. Their genetic modification capabilities enable the replication of gene mutations found in patients, facilitating the study of mutation function. Furthermore, iPSCs can be modified to develop allogeneic off-the-shelf immunotherapy drugs, evading allogeneic immune rejection and reducing the risk of graft-versus-host disease (GvHD). The application of iPSCs in tumour immunotherapy presents opportunities for the development of personalized treatments, but also comes with challenges that need to be addressed. We summarized the published pre-clinical and clinical studies of iPSC in tumour immunotherapy in Table 1 and discussed them in detail in the following.

**Table 1.** Summarization of the process of iPSCs’ utilization in tumour immunotherapy

Stages	Key points	Detail factors
Induction	Starting cells	Blood cells, fibroblasts, endothelial cells and so forth
	Generation technology	Ectopic expression of transcription factors, episomal plasmid, mRNA, micro-RNAs, proteins and small molecules
	Gene editing	Gene correction, gene insertion, gene knockdown and gene activation
Differentiation	Progenitor cell differentiation	Haematopoietic progenitor cell for immune cell differentiation
		Tissue-specific progenitor cell for tumour vaccine
	Target cell differentiation	Immune cells: T cells, NK cells, macrophages and DCs
		Tissue-specific tumour cell
	Target cell characterization	Purity and function
Application	Immune cell therapy	CAR or TCR-T/NK/macrophage, antigen presentation and so forth
	Cancer vaccine	Activation of the receptor’s immune response to the tumour

### iPSC-derived NK cells

NK cells that mature in the bone marrow are the first defence line of our body. In the realm of traditional NK cell immunotherapy research, the sources of NK cells have primarily been peripheral blood, umbilical cord blood and immortalized cell lines. While umbilical cord blood contains a higher proportion of NK cells compared to peripheral blood, obtaining a sufficient quantity for cell therapy remains challenging (Ref. 8). Immortalized NK cell lines, on the other hand, often exhibit limitations, such as the absence of CD16 for mediating antibody-dependent cytotoxicity or weakened cytotoxicity due to irradiation before patient transfer (Refs. 9, 10). In light of these challenges, iPSCs have emerged as a promising source of NK cells. By deriving iPSCs from a small number of somatic cells and subsequently differentiating them into NK cells, the cost and labour associated with autologous NK cell therapy can be significantly reduced (Refs. 11, 12).

Over the past decade, significant progress has been made in the differentiation of iPSCs into NK cells. Drawing from the methodology used for ESCs differentiation, iPSCs differentiation to NK cells (iNK) has advanced considerably (Ref. 13). Initially, iPSCs differentiation relied on mouse stromal cell lines as feeder cells, with the process taking ~2 months (Ref. 14). However, concerns regarding unknown factors secreted by the feeder cells prompted a shift towards feeder-free methods. A breakthrough came in 2013 when a new approach involving the formation of embryoid bodies (EBs) without feeder layer cells, followed by cytokine-directed differentiation, was proposed (Ref. 15). This method eliminated the reliance on feeder layer cells and opened up possibilities for the mass production of differentiated NK cells. In order to keep the consistency between batches, the number of iPSCs and the speed of centrifugation used to form EBs should be constant. Subsequent advancements in 2019 introduced the use of Rho-related protein kinase inhibitors, resulting in shortened differentiation times and increased efficacy. Additionally, a separate protocol demonstrated the differentiation of iPSCs into a mixed cell population of NK/innate lymphoid cells, showing promising results in a mouse model of ovarian cancer (Ref. 16). These findings have paved the way for the clinical use of iPSC-derived NK cells, offering potential for therapeutic applications.

Compared to traditional sources of NK cells, iNK cells offer a homogeneous and highly standardized alternative that is convenient for gene editing. Unlike blood NK cells, which can vary between donors and even within the same donor at different stages, iNK cells maintain high consistency due to the controlled iPSC characteristics and differentiation conditions. Additionally, gene editing of iNK cells can be performed during the iPSC stage, making it easier to operate compared to directly editing primary NK cells. This allows for the passage of edited genes to differentiated cells, resulting in homogenous engineered NK cells across multiple batches (Ref. 17). Pre-clinical and clinical trials implementing iPSCs, including iNK, are currently underway for various diseases (Ref. 18). The most common use of iPS-NK cells is in the form of iPS-CAR-NK or CAR-iNK against cancer, such as CD276-targeted CAR-iNK cells against human oesophageal squamous cell carcinoma and MUC1-targeted CAR-iNK cells against human tongue squamous cell carcinoma. Both of them have demonstrated effective anti-tumour efficacy (Refs. 19, 20). In addition to the direct cytotoxicity to tumour cells, iNK can also recruit T cells into tumour tissues and, thus, enhance the efficacy of anti-PD1 therapy (Ref. 21). However, it is important to note that iNK cells have disadvantages as well. Researchers have found that due to immaturity and imbalance of activating and inhibitory NK cell receptors, iNK cells are sensitive to the cytotoxic potential of autologous NK cells, leading to reduced therapeutic effect and short internal duration of iNK cells *in vivo* (Ref. 22).

### iPSC-derived T cells

T cells, matured in the thymus, are the main ingredient of lymphocytes. Currently, CAR-T cell therapies approved for commercial use have demonstrated significant efficacy in treating haematological malignancies, particularly in patients with advanced-stage tumours that have relapsed or are refractory to other treatments. These therapies typically utilize the patient’s own T cells as a starting material. However, autologous T cells present challenges, such as manufacturing delays, high production costs, standardization difficulties and potential production failures due to T-cell dysfunction in the patient. For solid tumours, CAR-T cell therapies are not as effective as in haematological malignancies because the acidic and hypoxic microenvironment inhibits the expansion and persistence of most T cells. Recently, researchers found that central memory T cells (TCMs) have superior expansion and persistence ability in a solid tumour environment. Stem cell memory T cells are even more effective than TCMs in solid tumours (Refs. 23–25). Therefore, iPSCs are considered a good candidate for solving these problems.

The differentiation of T cells from iPSCs presents a promising avenue akin to the success of iNK. However, it is noteworthy that the differentiation process of iPSCs to T cells is notably more intricate and time-consuming compared to iNK cells. Both methods necessitate EB formation and haematopoietic progenitor cell (HPC) differentiation as initial steps (Ref. 26). Notch signalling transduction plays a pivotal role in the differentiation and maturation of T cells from HPCs, with Delta-like ligand 4 shown to induce high-level Notch signalling crucial for desired T-cell production (Ref. 27). Cytokines, such as Flt3L, interleukin (IL)-7 and OP9-DL1, are also essential in the differentiation process, leading to the production of pro-T cells that can be activated to obtain mature T cells (Ref. 28). The use of artificial thymus organs, simulating the thymic environment, provides a unique innovation for T-cell differentiation, offering a flexible approach for iPSCs to differentiate into mature T cells (Refs. 29, 30). Furthermore, the removal of feeder cells and foetal bovine serum during the differentiation process for clinical immunotherapy is imperative. Researchers incorporate Notch ligands into the culture system using proteins as carriers to avoid feeder layer cells (Ref. 31). Challenges also exist regarding the random T-cell receptors (TCRs) of iPSC-derived T cells (iT), prompting the induction of T-iPSCs from donor effector T cells to ensure similar TCR expression (Refs. 28, 32). Alternatively, the ectopic expression of TCR genes in iPSCs through gene-editing methods has been explored to generate tumour antigen-specific cytotoxic T lymphocytes (CTLs) (Refs. 33–35). As research progresses, simplifying the process of T-cell differentiation and improving the function remain imperative for iT cells’ large-scale clinical applications (Ref. 36).

To avoid the immune rejection of allogenic iPSC-derived CAR-T cells, also known as CAR-iT, researchers have deleted β2-microglobulin, class-II major histocompatibility complex (MHC) trans activator and NK cell-ligand poliovirus receptor CD155, while overexpressing single-chain MHC class-I antigen E in iPSCs. These modifications allow the resulting CD20 CAR-iT cells to evade recognition by various immune cells and maintain anti-tumour potency in pre-clinical models. To enhance the proliferation and persistence of effector T cells within solid tumours, researchers knock out the diacylglycerol kinase gene and transduce genes encoding membrane-bound IL-15 and its receptor subunit IL-15Rα to CAR-iT cells. These engineered CAR-iT cells have demonstrated therapeutic outcomes comparable to primary CD8^+^ T cells bearing the same CAR in multiple animal models with tumours. These advancements hold promise for improving the effectiveness of CAR T-cell immunotherapies against solid tumours (Refs. 37, 38). Additionally, to prevent GvHD, researchers deleted TCR by knocking out the TCR α constant gene and found that no GvHD occurred (Refs. 39, 40).

In light of current advancements, it is evident that iPSCs-T, when coupled with CARs (CAR-iT), are still in the nascent stages of development and warrant additional pre-clinical and clinical investigations (Ref. 41).

### iPSCs-derived macrophages

Macrophages play a crucial role as major innate immune cells, contributing to various functions in immunity, inflammation and tissue repair. Their significant phagocytic ability, antigen-presenting activity and secretion of cytokines and chemokines enable them to effectively infiltrate dense tissues and accumulate in tumours. In fact, macrophages represent the largest population of immune infiltrates in solid cancers, constituting nearly 50% of the cell mass in most cases (Ref. 42). These characteristics position macrophages as promising candidates for manipulation in tumour immunotherapy (Ref. 43). However, despite the progress made in macrophage immunotherapy, there are notable concerns, such as limited cell resources, resistance to gene transfer and potential inflammatory pathology, which currently hinder their application as potent cancer immunotherapy (Ref. 42). The integration of iPSCs preparation and genetic editing technology offers the potential for solving these problems and developing next-generation macrophages with specific tumour antigen recognition units, feasible genetic modification and enhanced expansion capability (Refs. 44, 45).

As mature tissue-resident macrophages are derived from yolk-sac (YS) macrophages that arise from YS progenitors during embryogenesis, iPSCs need to undergo a process similar to YS-haematopoiesis to get functional macrophages *in vitro.* Differentiation protocols commonly utilize EB-based or monolayer-based methods, with studies indicating that the monolayer-directed approach yields a significantly higher quantity of macrophages compared to the EB-based method (Refs. 46, 47). These methods differentiated macrophages are primary macrophages, and they can terminally differentiate into specialized mature macrophages *in vivo* or with the help of organ cues *in vitro* (Ref. 46).

Macrophages have shown promise as targets for CAR technology due to their potential to infiltrate and influence solid malignancies (Ref. 48). A notable development in this area is the CAR-expressing macrophages (CAR-iMacs) technology platform derived from iPSCs, which confers antigen-dependent macrophage functions and demonstrates anti-tumour activity *in vivo* (Ref. 49). Before the usage of CAR-iMacs in tumour immunotherapy, researchers should guarantee that these CAR-iMacs are M1 subtype and have pro-inflammatory responses, as most of the primary macrophages that are derived from iPSCs are M2 subtype. The direct method to convert M2 macrophages to M1 subtype is stimulation by lipopolysaccharide (LPS) or interferon-γ *in vitro.* One study showed that Anti-CD19-CAR-iMacs exhibit enhanced and antigen-dependent phagocytosis, with increased pro-inflammatory responses when co-cultured with CD19^+^ tumour cells or tumour cells from patients with leukaemia (Ref. 50). Additionally, another research demonstrated that ACOD1-depleted CAR-iMacs showed enhanced capacity in repressing tumours and increased survival in ovarian or pancreatic cancer mouse models, especially when combined with immune checkpoint inhibitors (ICIs) (Ref. 51). Mechanistically, depletion of ACOD1 reduces levels of the immuno-metabolite itaconate and allows KEAP1 to prevent Nuclear factor erythroid 2-related factor 2 (NRF2) from entering the nucleus to activate an anti-inflammatory programme. Furthermore, another research group improved the structure of CAR with the addition of a Toll-like receptor 4 (TLR4) intracellular Toll/IL-1R (TIR) domain in the intracellular activation domain (Ref. 52). Mechanistically, once stimulated by LPS, TLR4 interacts with adaptor molecules via its TIR signal transduction domain and leads to nuclear translocation of nuclear factor kappa B/p65, promoting the expression of pro-inflammatory cytokines (Ref. 53). Researchers found that introducing this domain into the CAR would increase the M1-like phenotype of iMacs upon engaging antigens and improve anti-tumour efficacy (Ref. 52).

### iPSC-derived DCs

DCs derived from the bone marrow play a crucial role as the primary antigen-presenting cells within the immune system, facilitating the initiation and coordination of immune responses (Ref. 54). These cells are heterogeneous in nature and can be broadly categorized into different groups: conventional or classical DCs, plasmacytoid DCs, inflammatory DCs, and Langerhans cells (Ref. 55). DCs have extracellular and intracellular pattern recognition receptors that can identify danger signals. After sensing danger signals, DCs can be activated to endocytic or phagocytic antigens and present them to both CD4^+^ and CD8^+^ T cells. The activated DCs can also secrete cytokines and chemokines to recruit T cells. These features enable DCs to be utilized in the development of immunogenic vaccines for targeted disease treatments, such as cancer (Refs. 56, 57). In the field of cancer treatment, DCs loaded with tumour-specific antigens are utilized as a form of cancer immunotherapy. In 2010, the US Food and Drug Administration approved Sipuleucel-T (Provenge®), the DC-based vaccine to treat prostate cancer patients. It has increased the average overall survival of hormone-refractory prostate cancer patients for about 4 months (Ref. 58). Besides prostate cancer, lots of clinical trials of DCs vaccines are also proceeding on many other tumour types, such as ovarian cancer, gastric cancer lung cancer and so forth.

However, challenges in sourcing DCs from conventional origins, such as the bone marrow, peripheral blood and cord blood, have significantly impeded their routine application (Ref. 59). With high differentiation ability, iPSCs offer a promising alternative for generating ample quantities of DCs (iDCs) suitable for basic and preclinical studies (Ref. 60). As DCs are haematopoietic cells, the initial differentiation of DCs from iPSCs involves the generation of HPCs. After HPCs, many different cytokines are used to stimulate DC specification and maturation, such as basic fibroblast growth factor, bone morphogenic protein 4, fms-related tyrosine kinase 3 ligand, stem cell factor, TLR, tumour necrosis factor alpha, thrombopoietin and so forth. OP9 cells or Matrigel is used to coculture with the differentiated cells. Therefore, various research groups have endeavoured to establish DC differentiation protocols from human iPSCs, with differences in timelines, feeder/Xeno-free conditions and yields among these protocols (Refs. 61–71).

The iDC-based vaccines also showed efficacy in cancer treatment. For example, fully matured iDCs expressing tumour-associated antigens have been shown to present specific peptides to MHC I and induce CTL stimulation in immunized mice (Ref. 72). Another study has indicated that granulocyte–macrophage colony-stimulating factor producing iDCs can suppress myeloid-derived suppressor cells and facilitate a CTL-mediated anti-tumour response (Ref. 73). Recent research has shown that, in combination with local radiotherapy, in situ-delivered iDCs exhibit an enhanced ability to migrate to tumour-draining lymph nodes, interact with T cells, promote CTL infiltration and sensitize programmed death ligand-1 blockade. This result suggested that the iDC vaccine can combine with radiotherapy and ICIs to improve anti-tumour efficacy (Ref. 74).

While human-engineered iDCs have achieved progress in pre-clinical settings, there are still some concerns that need to be addressed. The first one is to increase differentiation efficacy; the development of bioinformatic methods and information generated by single-cell transcriptomics and proteomics can help the discovery of important molecules in the differentiation of DCs, and the usage of these molecules may increase the yield of iDCs. The second one is to decrease the cost of producing iDCs. The replacement of cytokines with small chemical molecules can reduce the costs of the differentiation process.

### iPSC-derived cancer vaccines

Besides iDC-based cancer vaccines, iPSCs alone can also be used as cancer vaccines, as previous studies have identified numerous similarities between iPSCs and tumour cells, including the capacity for self-renewal and infinite cellular proliferation, high telomerase activity promoting telomere elongation and a metabolic pattern characterized by glycolysis in response to rapid proliferation (Ref. 75). The four Yamanaka transcription factors (Oct4, Sox2, Klf4 and c-Myc) that are used in iPSC induction contribute to the carcinogenesis of iPSCs. Overexpression of Oct4 can induce miR-125b expression to prevent tumour cell apoptosis and maintain the stemness of many types of cancer cells (Refs. 76, 77). Sox2 can form a heterodimer with Oct4 and downregulate the expression of CDX2 to help maintain the stem-like state of tumour cells (Ref. 78). Klf4 can also help convert cancer cells into a stem-like state through increasing the expression of E-cadherin (Ref. 79). c-Myc is a well-defined oncogene.

While recent clinical results have shown promise for cancer cell vaccines in treating cancer, their efficacy against established tumours remains limited, and personalized vaccines based on a patient’s unique neoantigens can be time-consuming to produce. Patient somatic cell-derived iPSCs can be used as personalized cancer vaccines both in prevention and in treatment. In 2018, Kooreman et al. made a significant advancement by identifying that vaccination of mice with iPSCs induces prophylactic and therapeutic anti-cancer immunity to shared antigens, suggesting a potential avenue for the rapid development of iPSC-based personalized cancer vaccines (Ref. 80). Later studies have improved the therapeutic efficacy of iPSC-based cancer vaccines in different ways. For instance, neoantigen-engineered iPSC cancer vaccines have demonstrated the ability to trigger neoantigen-specific T-cell responses and improve the therapeutic efficacy of radiotherapy in poorly immunogenic colorectal cancer and triple-negative breast cancer (Ref. 81). Additionally, researchers have explored the use of iPSC-differentiated tumour cells as a potential tumour vaccine, achieving promising results in orthotopic mouse models of pancreatic and lung cancer (Refs. 82, 83).

### Opportunities and challenges

**Figure 3.**
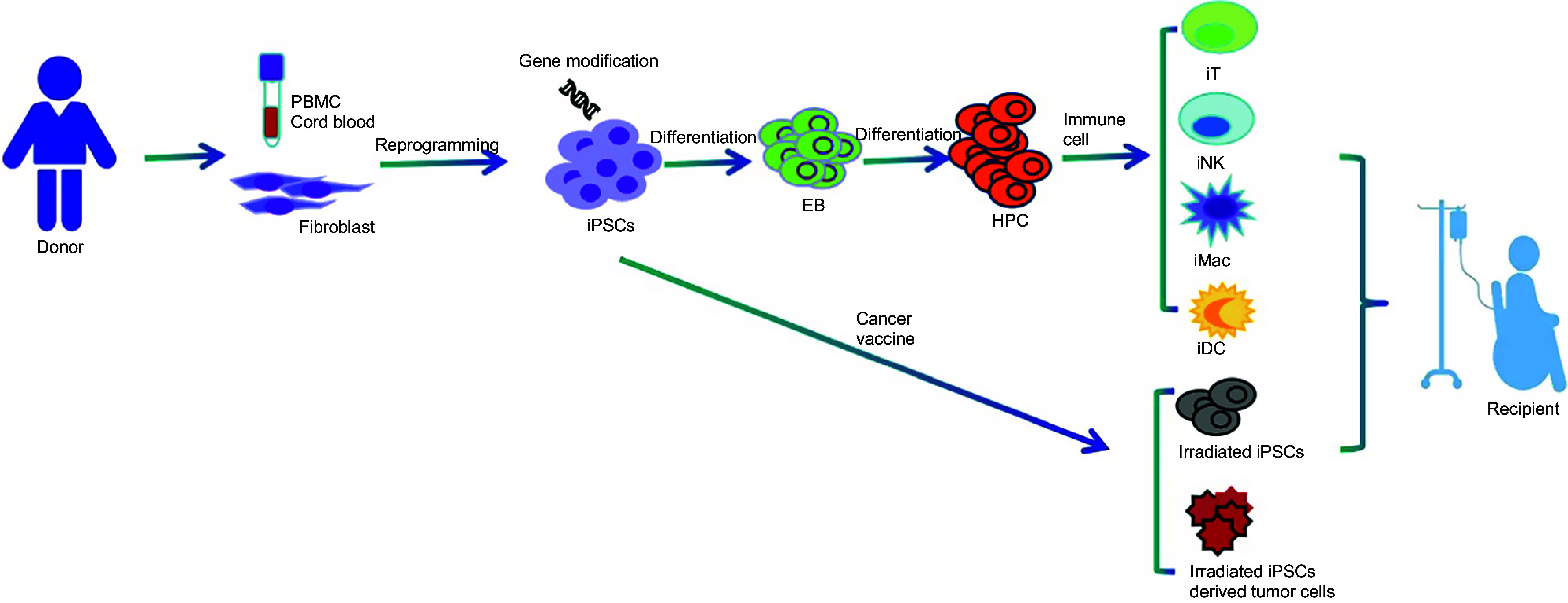
Overview of iPSC-derived immunotherapy.

The utilization of autologous immune cells in cancer therapy is widely known to be both costly and time-consuming, posing challenges for patients who require immediate treatment. Fortunately, the combination of iPSC technology with gene-editing technology holds great promise in addressing these issues by enabling the production of allogenic off-the-shelf immune cell products, as illustrated in Figure 3.

With the development of iPSC technology, the reprogramming of somatic and cancer cells into iPSCs has become increasingly feasible. The successful application of small-molecule cocktails during the inducing process further enhanced the safety and clinical potential of iPSCs (Ref. 84). These also led to the establishment of numerous iPSC banks in different regions. Researchers collected human leukocyte antigen homozygous donors’ somatic cells that cover the largest population to construct iPSC banks (Ref. 85). Cells in these banks can be used as starting cell sources to develop allogeneic cell products and are widely used in regenerative medicine, disease modelling and drug discovery (Refs. 86–89).

Although iPSCs conquer the ethical problems faced by ES cells, their clinical application still faces regulatory challenges. Based on the process for the generation of iPSCs and differentiation into target cells, the regulatory process should be divided into four stages (Ref. 90]. The first stage is starting the cell source. Different starting cells have different features and differentiation preferences; therefore, researchers should choose specific starting cells based on their objectives. The second stage is the reprogramming process. In this stage, researchers should focus on the reprogramming objectives and techniques used for reprogramming. Different reprogramming targets should choose different reprogramming techniques. The third stage is expansion and banking. In this stage, researchers should characterize the induced cells and ensure their stability. The last stage is the final product characterization. Researchers should guarantee the identity, stability, activity/potency and safety of the final product. The most important safety concern is tumorigenicity. Several reasons cause tumorigenicity; one of them is the low differentiation efficiency of iPSCs, which leads to the presence of undifferentiated or immature cells in the final products, and uncontrolled proliferation of these cells can lead to tumour formation. Reprogramming factors used in iPSC production have also been found to improve tumorigenesis as they activate oncogenes during the induction of pluripotency. The unpredictable genetic changes in iPSCs during the reprogramming or differentiation process further increased the risk of tumorigenicity. To alleviate this risk, researchers should consider the introduction of a suicide switch system, such as Herpes simplex virus thymidine/Ganciclovir and inducible caspase9 to iPSCs to prevent tumorigenesis.

As discussed previously, lots of work still needs to be done before iPSC-derived final cell products can be widely adopted for clinical application. Fortunately, the establishment of iPSC banks has alleviated the regulatory challenge in the first three stages if researchers choose cells from these banks.
